# Osteoblast-Specific Overexpression of Nucleolar Protein *NO66*/RIOX1 in Mouse Embryos Leads to Osteoporosis in Adult Mice

**DOI:** 10.1155/2023/8998556

**Published:** 2023-01-10

**Authors:** Qin Chen, Krishna M. Sinha, Benoit de Crombrugghe, Ralf Krahe

**Affiliations:** ^1^Department of Epigenetics and Molecular Carcinogenesis, The University of Texas MD Anderson Cancer Center, Houston, TX 77030, USA; ^2^Department of Genetics, The University of Texas MD Anderson Cancer Center, Houston, TX 77030, USA; ^3^Clinical Cancer Prevention, The University of Texas MD Anderson Cancer Center, Houston, TX 77030, USA

## Abstract

In previous study, we showed that nucleolar protein 66 (NO66) is a chromatin modifier and negatively regulates Osterix activity as well as mesenchymal progenitor differentiation. Genetic ablation of the *NO*66 (*RIOX1*) gene in cells of the *Prx*1-expressing mesenchymal lineage leads to acceleration of osteochondrogenic differentiation and a larger skeleton in adult mice, whereas mesenchyme-specific overexpression of *NO*66 inhibits osteochondrogenesis resulting in dwarfism and osteopenia. However, the impact of NO66 overexpression in cells of the osteoblast lineage *in vivo* remains largely undefined. Here, we generated osteoblast-specific transgenic mice overexpressing a FLAG-tagged NO66 transgene driven by the 2.3 kB *alpha*-1*type I collagen* (*Col1a*1) promoter. We found that overexpression of *NO66* in cells of the osteoblast lineage did not cause overt defects in developmental bones but led to osteoporosis in the long bones of adult mice. This includes decreased bone volume (BV), bone volume density (bone volume/total volume, BV/TV), and bone mineral density (BMD) in cancellous compartment of long bones, along with the accumulation of fatty droplets in bone marrow. *Ex vivo* culture of the bone marrow mesenchymal stem/stromal cells (BMSCs) from adult *Col1a1*-NO66 transgenic mice showed an increase in adipogenesis and a decrease in osteogenesis. Taken together, these data demonstrate a crucial role for *NO66* in adult bone formation and homeostasis. Our *Col1a1*-NO66 transgenic mice provide a novel animal model for the mechanistic and therapeutic study of NO66 in osteoporosis.

## 1. Introduction

In mammals, flat and long bones form through intramembranous and endochondral ossification, respectively. In intramembranous ossification, mesenchymal stem cells differentiate directly into cells of the osteoblast lineage, including preosteoblast, immature osteoblast, mature osteoblast, and osteocyte. In endochondral ossification, mesenchymal stem cells differentiate into osteochondroprogenitors, which then segregate into chondrocytes that form a cartilage template or osteoprogenitors that differentiate toward the osteoblast lineage to form bone tissue. Maintenance of healthy bone mass depends upon constant homeostasis, a balance between osteoblast-mediated bone formation and hematopoietic osteoclast-mediated bone resorption. Imbalance between these two processes results in osteoporosis, which is a common bone disease in the aging population and especially in postmenopausal women [1]. Another mechanism of osteoporosis involves dysregulated osteogenesis and/or adipogenesis from bone marrow-derived mesenchymal stem/stromal cells (BMSCs), which have the capacity to differentiate into both osteoblastic and adipocytic lineages [2, 3]. In aging patients with osteoporosis, the decrease in bone formation often coincides with the increase in marrow adiposity [4, 5]. However, it remains unclear how these two processes are coupled in the bone marrow.

Commitment and differentiation of mesenchymal stem cells or BMSCs into the osteoblastic lineage are tightly controlled by specific signaling molecules including bone morphogenetic protein 2 (BMP2) and insulin-like growth factor 1 (IGF1), as well as essential bone-forming transcription factors, such as runt-related transcription factor 2 (Runx2) and Osterix (Osx) [6–8]. In mice with a germline deletion of the *Osx* (*Sp*7) gene, formation of both endochondral and intramembranous bones was completely abolished [6]. The activity of Osx can be regulated by the nucleolar protein 66 (NO66), also known as Ribosomal Oxygenase 1 (RIOX1). We previously showed that NO66 binds to Osx and inhibits the transactivation of its target genes *bone sialoprotein* (*Bsp*) (*Ibsp*) and *Osteocalcin* (*Oc) (Bglap*) [9]. In addition to inhibiting Osx activity, NO66 also negatively regulates osteogenic differentiation of mesenchymal stem cells both *in vitro* and *in vivo* [9–11]. In the cultured pluripotent mesenchymal precursor C2C12 cell line, overexpression of NO66 inhibits osteogenic differentiation and expression of the osteoblast-differentiation marker gene [9]. In transgenic mice, overexpression of NO66 in cells of *Prx*1-expressing mesenchymal origin inhibits osteogenesis and reduces the number of Osx-expressing preosteoblasts and osteoblasts, concurrent with the reduction in IGF1R/Akt signaling and expression of bone formation marker genes, leading to both growth retardation and osteopenia phenotype [11]. Conversely, depletion of NO66 in cells of Prx1-expressing mesenchymal origin increases the number of preosteoblasts and osteoblasts upregulates expression of bone-forming signal molecules and marker genes, including *Bmp*2, *Igf*1, Osx (*Sp*7), *type I collagen a1* (*Col1a1*), and *Bsp (Ibsp)*. As a result, NO66-null mice exhibit an acceleration of skeletogenesis as well as bone formation [10], demonstrating an essential role for NO66 in commitment and osteogenic differentiation of mesenchymal stem cells. Moreover, our previous *in vitro* studies showed that NO66 is a negative regulator of osteoblast differentiation [9, 12]. Knockdown of NO66 in mouse preosteoblast MC3T3-E cell line accelerates the BMP2-induced osteoblast differentiation and maturation [9]. However, it remains unclear whether NO66 is important in osteoblast differentiation and maturation *in vivo*.

Here, we generated transgenic mice overexpressing a FLAG*-tagged* NO66 transgene driven by the 2.3-kb *Col1a1* promoter to study the *in vivo* role of NO66 in osteoblastogenesis. Previous studies indicated that this promoter is highly active in the osteoblast lineage of mouse embryos beginning around embryonic day 14.5 (E14.5) [13, 14]. We found that osteoblast-specific overexpression of NO66 shows minimal alteration in the formation of developmental bone. In contrast, it affects the homeostasis of mature bones, including the impaired formation of cancellous bone and increased accumulation of bone marrow fat (BMF), the predominant feature of osteoporosis.

## 2. Materials and Methods

### 2.1. Generation and Genotyping of *Col1a1*-*NO*66 Mice

All experimental procedures involving animals complied with the National Institutes of Health guidelines for the care and use of laboratory animals and were approved by the Institutional Animal Care and Use Committee of the University of Texas MD Anderson Cancer Center (IACUC Protocol No: 108807638).

**Figure 1:**
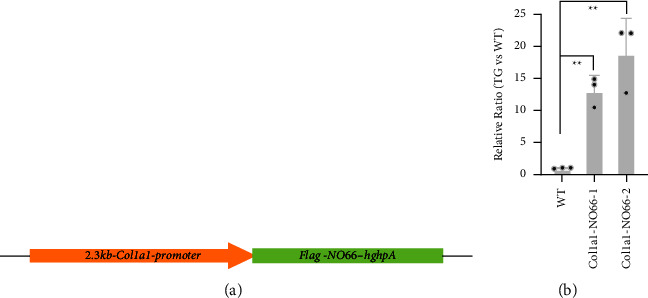
Generation of *Col1a1-NO*66 transgenic (TG) mice. (a) Schematic of the *Col1a1-NO*66 construct for microinjection to generate transgenic mice. (b) The results of RT-QPCR assay for mRNA expression of *NO66 gene* (^*∗∗*^*p* < 0.001).

Generation and genotyping of the *Col1a1-NO*66 transgenic mice were performed, as previously described [11]. Briefly, a FLAG-tagged cDNA [9] was subcloned downstream of the 2.3 kB *Col1a1*-promoter in a pBluescript SK vector, to generate a new *Col1a1-NO66* construct (Figure 1(a)). Plasmid DNA of this construct was microinjected into fertilized B6D2 F1 eggs, which were then transferred into CD1 foster mothers. Transgenic offspring were crossed three times with wild-type (WT) C57BL/6 mice to establish transgenic lines for the study. The founders and offspring of transgenic mice were genotyped by PCR assays using tail DNA and *Hgh* primers: 5′-TGTCTGACTAGGTGTCCTTC;5′-GCAAGCAACTCAAATGTCC, as described previously [11].

### 2.2. RNA Extraction and RT-qPCR Assay

RNA extraction and real-time quantitative polymerase chain reaction (RT-qPCR) were performed as previously described [10, 11]. Briefly, total RNA of mouse calvaria at embryonic day 18.5 (E18.5) was isolated using TRIzol reagent (Invitrogen) according to the manufacturer's protocol. Total RNA was pretreated with TURBO DNase (Ambion) to remove genomic DNA contamination and then reverse-transcribed into cDNAs using a high-capacity reverse transcription kit (Applied Biosystems). For RT-qPCR assay, 50 ng cDNA and a gene-specific TaqMan primer-probe, including mouse *NO66*, *Bsp*, *Col1a1,* and *Alp* (Applied Biosystems) were used in each PCR reaction (triplicate). Levels of mRNA expression were normalized relative to the *Hprt* housekeeping gene.

### 2.3. Histological Analysis

Limbs of E15.5, E18.5 mouse embryos and pups at postnatal day 4 (P4) and P10, as well as decalcified femurs of adult mice at 3-months (3 m) or 4m of age were paraformaldehyde-fixed, paraffin-embedded, sectioned at 7 *μ*m thickness, and stained with hematoxylin and eosin (H&E) following routine experimental procedures [10, 11]. Mineral deposition in embryonic bones was examined by Alizarin red and von Kossa's staining, as described previously [6, 10, 11].

### 2.4. Immunostaining

The FLAG-tagged NO66 protein was examined by immunostaining as described previously [11]. In brief, paraffin-embedded mouse femoral sections from embryos (E15.5 and E18.5) or postnatal pups (P10) were deparaffinized and subjected to enzymatic digestion with hyaluronidase (2 mg/mL in phosphate buffer, pH 5.5 (MP Biomedicals). Primary antibody against the FLAG epitope (1 : 200; Chemicon) was incubated overnight at +4°C. The secondary antibodies, including 555 goat antimouse (1 : 1000; Molecular Probes/Invitrogen) or goat antimouse IgG linked with HRP (1 : 1000; Abcam), were incubated for 1 hr at room temperature. For immunofluorescence staining, slides were washed and mounted with antifade-Gold with 4′, 6-diamidino-2-phenylindole (DAPI; Molecular Probes) and then analyzed under a fluorescence microscope. For immunohistochemistry (IHC) staining, slides were washed and then stained using a DAB substrate kit (Vector Laboratories) following the manufacturer's protocol.

### 2.5. Specimen CT (SP-CT) Scanning

Femurs of mice at three-month (3 m) old were isolated and fixed in 4% paraformaldehyde overnight at room temperature and then stored in 70% ethanol for SP CT scanning (GE Medical Systems, London, Ontario). Scanning and analyses of bone images were conducted in the Small Animal Imaging Facility at the University of Texas MD Anderson Cancer Center, as described previously [10, 15].

### 2.6. Isolation and Culture of Bone Marrow Stromal Cells (BMSC)

Primary culture of BMSC was performed following the procedures described previously [16] with minor modifications. Briefly, femoral and tibia bones were isolated from 3 m old nontransgenic and *Col1a1-NO66* transgenic mice. After removing connective tissues, bones were kept in ice-cold PFA solution containing 1 × PBS (without Ca^2+^ and Mg^2+^)/2% FBS (v/v)/1 × Pen/Strep antibiotics. Bone marrow was flushed out using 4-5 ml of alpha-MEM supplemented with 5% FBS and 1 × Pen/Strep antibiotics (using a 25 G needle) and transferred into a 50 ml tube on ice. The bone marrow plugs were homogenized by repeated passage through a 21 G needle, mixed 1 : 1 with distilled sterile water for 2 min to lyse erythrocytes, and then centrifuged at 380 × g for 5 min. The cell pellet was resuspended in complete culture medium (alpha-MEM, 20% FBS, 2 mM L-glutamine, 100 U/ml of Pen/Strep), and cultured in a 37°C humidified incubator containing 3% O_2_, 5% CO_2_, and 90% N_2_. The culture medium was changed every other day. Upon reaching 80% confluence, cells were trypsinized and replated for further analysis.

For osteogenic differentiation, cells were seeded at a density of 15 × 10^3^ cells/cm^2^ in normal culture medium. On day six, the culture medium was replaced with osteogenic medium containing the complete alpha-MEM supplemented with 10 mM sodium *β*-glycerophosphate, 10 nM dexamethasone, and 50 *μ*g/ml ascorbic acid. To initiate adipogenic differentiation, the cells were cultured in complete alpha-MEM supplemented with 5 *μ*g/ml insulin, 1 *μ*M rosiglitazone, and 1 *μ*M dexamethasone [17].

### 2.7. Statistical Analysis

All statistical results are presented as the mean ± standard error (SE). The differences between groups were calculated using the two-tailed Student *t-*test and *p* values <0.05 were considered statistically significant.

## 3. Results

### 3.1. Generation of Osteoblast-Specific* NO66* Transgenic Mice

**Figure 2:**
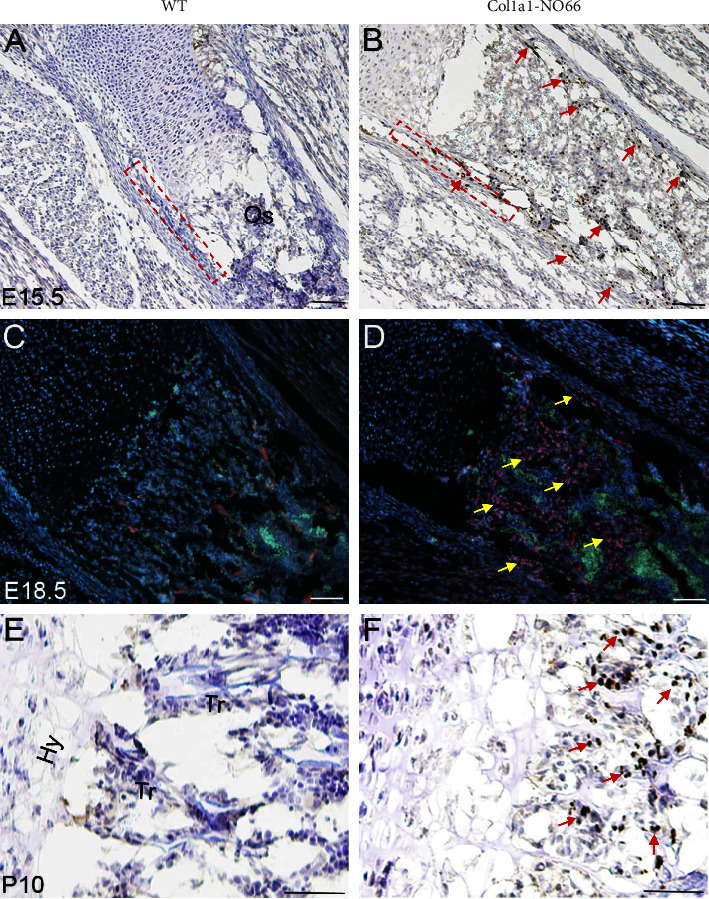
Immunostaining. (a), (b) (e), (f), immunohistochemistry. The femur sections of wild type (WT) (a), (e) and *NO66* transgenic (TG) (b), (f) mice at E15.5 and P10 were stained with anti-FLAG antibody. The boxed area in (a) and (b) indicate periosteum. Red arrows in (b) and (f) point to cells expressing FLAG-tagged *NO66* transgenic protein (dark brown nuclear staining). Os, ossification center; Hy, hypertrophic chondrocytes; Tr, trabeculae. (c), (d), immunofluorescence staining. The femur sections of WT (c) and TG (d) embryos at E18.5 were stained with anti-FLAG antibody (red). Cell nuclei were counterstained with DAPI (blue). Autofluorescence signal (green) within the ossification center (Os) indicates bone marrow. Yellow arrows in (d) point to cells expressing FLAG-tagged *NO66* transgenic protein (red nuclear staining).

To examine the role of NO66 in mature osteoblasts *in vivo*, we generated transgenic mice overexpressing the FLAG-tagged NO66 in cells of the osteoblastogenic lineage driven by a 2.3-kb *type I collagen promoter (Col1a1)* (Figure 1(a)). In mice, the 2.3 kB *Col1a1* promoter is active around embryonic day 14.5 (E14.5) and specifically drives transgene expression at high levels in osteoblasts and odontoblasts [14, 18]. We performed pronuclear injections of the *Col1a1-*NO66 construct and generated two stable transgenic mouse lines, named *Col1a1-NO66-1* and *Col1a1-NO66-2*. To determine expression of NO66 transgene in membranous bone tissue, we performed RT-qPCR assays using total RNA of calvaria from E18.5 wild type (WT) control and transgenic embryos. We observed the high-level mRNA expression of NO66 in both transgenic lines compared with WT control embryos (Figure 1(b); *p* < 0.001). To examine expression of NO66 transgene in endochondral bone tissue, we performed immunostaining of femur sections of WT and transgenic mice using anti-FLAG antibody. In the femur sections of E15.5 and E18.5 transgenic embryos from both lines, we detected the FLAG-tagged NO66 transgenic protein in nuclei of cells located in the perichondrium/periosteum regions and ossification centers (S Figures 1B and 1D; Figures 2(b) and 2(d)) comparing with nontransgenic controls (S Figures 1A and 1C; Figures 2(a) and 2(c)). By postnatal day 10 (P10), the FLAG-NO66 transgenic protein was predominantly present in the trabecular area of femur sections of transgenic mice (Figure 2(f)). The expression pattern of NO66 transgene driven by the 2.3 kB *Col1a1* promoter coincided with the developmental appearance of cells in osteoblastogenic lineage, consistent with previous reports [13–15, 18].

### 3.2. Formation of Intramembranous and Endochondral Bones in *Col1a1-NO*66 Mice during Development

**Figure 3:**
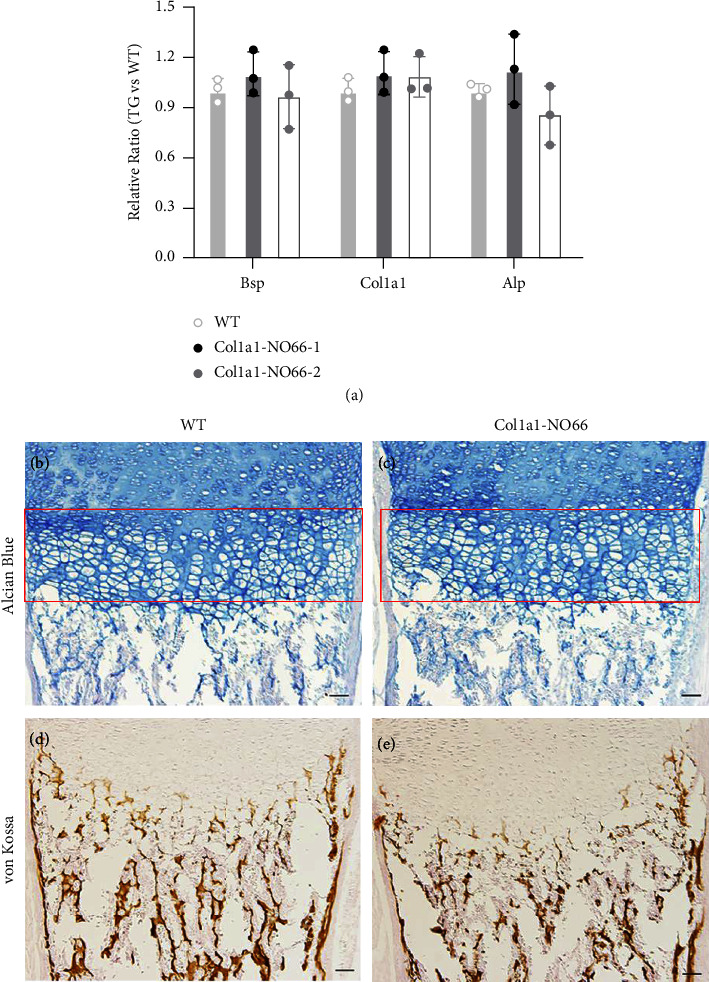
RT-qPCR and histological assays. (a). The results of RT-qPCR assay for expression of the bone-differentiation marker genes *Bsp*, *Col1a1,* and *Alp*. (b)–(e) Histological examination. Alcian blue (b), (c) and von Kossa (d), (e) staining of the distal femoral sections of newborn (P1) wild type (WT) and *NO66* transgenic mice. The red squares in (b) and (c) show hypertrophic zone of cartilage; brown staining signals in (d) and (e) indicate mineralized tissue.

Next, we sought to examine whether *NO66* overexpression in cells of the osteoblast lineage could affect intramembranous bone formation during early development. We performed RT-qPCR assays using total RNA of calvaria from E18.5 mouse embryos for changes in expression of bone-formation marker genes. We did not observe significant changes in the mRNA expression of *bone sialoprotein (Bsp)*, *Col1a1,* and *alkaline phosphatase (Alp)* in *NO66* transgenic embryos from bone lines when comparing with those of WT controls (Figure 3(a); *p* > 0.05). This suggests that *NO66* overexpression in cells of the osteoblast lineage may have minimal influence in the formation of intramembranous bones during embryogenesis.

We then examined the phenotypic changes in endochondral bones of mouse embryos and newborn mice. We performed histological examination of long bones of nontransgenic and *Col1a1-NO66* transgenic mice from both lines at different development stages. In femur sections of E15.5 *NO66* transgenic embryos, we did not observe any reduction in mineralized bone tissues marked by either von Kossa or Alizarin red staining when comparing with WT control embryos (S Figures 2A–2D). H&E staining of femur sections of newborn WT and transgenic mice also showed similar morphological appearance (S Figures 2E and 2F). When we stained the femoral sections of newborn WT and transgenic mice with Alcian blue (for cartilage) and von Kossa (for mineralized bone tissue), we also observed the similar staining pattern between the WT and transgenic mice (Figures 3(b)–3(e)). These data indicate that *NO66* overexpression in cells of the osteoblast lineage has minimal inhibitory effect on the formation of endochondral bones during embryonic and early mouse development.

### 3.3. Loss of Bone and Accumulation of Bone Marrow Fat in Adult *Col1a1*-*NO*66 Transgenic Mice

**Figure 4:**
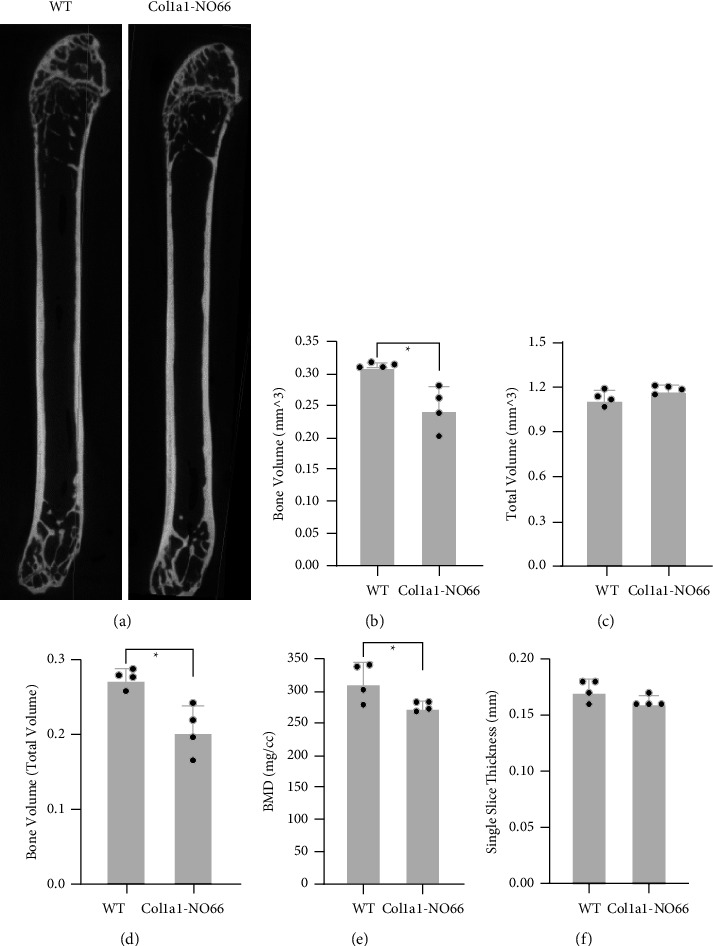
Specimen-computed tomography (SP-CT). (a), SP-CT scanning images of femurs from three-month-old female wild type (WT) and transgenic mice (*n* = 4). (b)–(f), Quantification of the SP-CT images shown in (a) (^*∗*^*p* < 0.05).

To investigate whether the osteoblast-specific overexpression of *NO66* could affect bone formation or homeostasis in adult mice, we first scanned the femurs of 3 m-old WT and *Col1a1-NO66* transgenic mice using specimen-computed tomography (SP-CT). The scanning results showed a significant decrease in bone mineral density and percentage of bone volume in the distal femurs of *NO66* transgenic mice when compared to WT controls (Figures 4(a)–4(e); *p* < 0.05). However, in the same transgenic mice we only observed a minimal reduction in the cortical bone thickness (Figure 4(f); *p* > 0.05). There was no significant difference in femur length between WT and transgenic mice (S Figure 3; *p* > 0.05).

**Figure 5:**
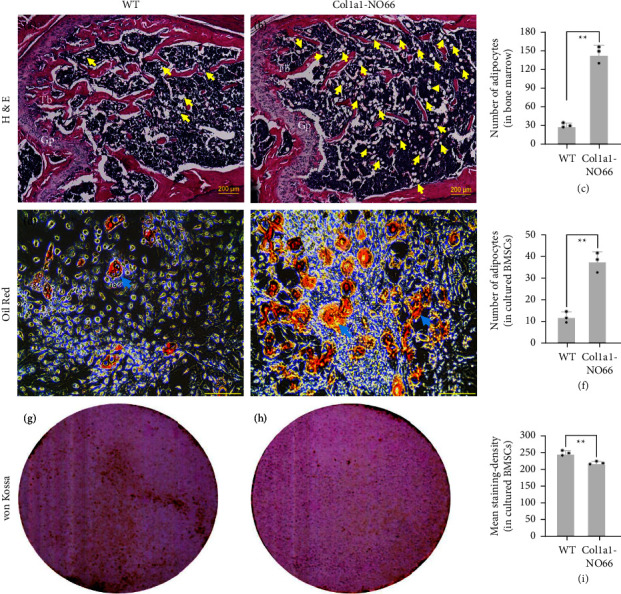
Histological examination and induction of adipogenic as well as osteogenic differentiation in *ex vivo* culture of bone marrow stromal cells (BMSC). (a), (b), H&E staining of the distal femur sections of three-month-old wild type (WT) and *Col1a1-NO66* transgenic mice. Yellow arrows indicate fatty droplets within the bone marrow. Gp, growth plate; Tb, trabeculae. (c) Number of fatty droplets shown in (a) and (b). (d), (e) (g), (h), Oil Red (d), (e), and von Kossa (g), (h) staining of the primary cultured BMSC from long bones of three-month-old female WT and transgenic mice (*n* = 5). Blue arrows in (d) and (e) point to fatty droplets. (f) Number of fatty droplets shown in (d) and (e) (counted within three randomly selected image-fields). (i) Quantification of staining density shown in (g) and (h) (using ImageJ software) (^*∗∗*^*p* < 0.001).

We then performed the histological examination of the long bones from 3 m to 4 m-old male and female mice. H&E staining of the femur sections of 4 m-old male transgenic mice revealed more empty-spaces and less marrow tissues than those in WT controls (S Figure 4). When we stained the femur sections of 3 m-old female WT and transgenic (from both lines) mice, we observed fewer trabeculae underneath their growth plates than those in the WT control mice (S Figures 5A–5C and Figures 5(a) and 5(b)). Interestingly, we also observed a markedly increased number of air-filledballoon-like cells (or empty cells), which implicate adipocytes (identified by yellow arrows) [19], in the bone marrow of transgenic femur sections when compared with WT controls (S Figures 5D–5F and Figures 5(a) and 5(b)). In comparison, the thickness of cortical bones around the middle shaft of those transgenic mice showed minimal alteration when compared with WT controls (S Figures 5G–5I), which was consistent with the SP-CT scanning results (Figure 4(f); *p* > 0.05). These observations suggested a loss of cancellous bone and a gain of bone marrow fat in adult mice with the osteoblast-specific overexpression of *NO66*.

### 3.4. *Ex vivo* Culture of BMSCs from Adult WT and *Col1a1*-*NO*66 Mice

**Figure 6:**
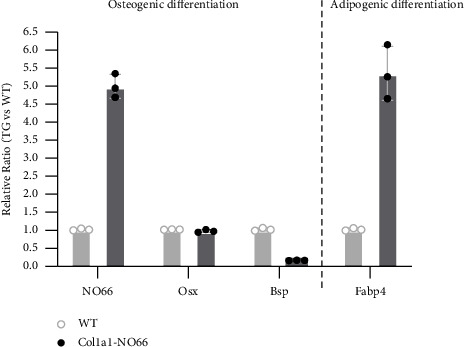
Gene expression analysis in BMSCs. Bar graph shows the RT-qPCR assay for expression of *NO66*, *Osx*, *Bsp,* and *Fabp4* genes in BMSCs cultured in osteogenic and adipogenic differentiation media.

BMSCs are multipotent adult stem cells that can differentiate into osteogenic and adipogenic lineages. The BMSCs-derived osteogenesis and adipogenesis often exhibit an inverse relationship in osteoporotic patients as well as animal models [20–23]. Therefore, we speculated that the inhibition of osteogenesis by osteoblast-specific overexpression of *NO66* might increase adipogenesis from the BMSCs of adult *Col1a1-NO66* mice. To test this hypothesis, we performed *ex vivo* culture of BMSCs from the 3 m-old WT and transgenic mice, and then induced the primary BMSCs to undergo adipogenic/osteogenic differentiation. After 7 days of adipogenic induction, Oil Red O staining showed more fatty droplets formed in BMSCs from *NO66* transgenic mice compared to that in WT controls (Figures 5(d)–5(f); *p* < 0.001). In contrast, osteogenic induction showed significant less mineralization in cultured transgenic BMSCs (Figures 5(g)–5(i); *p* < 0.001). To further confirm these observations, we examined mRNA expression of *NO66*, *Osx*, *Bsp,* and *Fabp4* in the primary cultured BMSCs after induction of osteogenesis or adipogenesis by RT-qPCR. In osteogenic-differentiated BMSCs derived from those *Co1a1-NO66* transgenic mice, we observed a high-level increase in expression of *NO66* and a marked decrease in expression of the bone-forming marker *Bsp*; however, we only observed a minimal alteration in expression of *Osx* (Figure 6), consistent with our previous study [9]. By comparison, in adipogenic-differentiated BMSCs expression of *Fabp4* (a marker of fat formation) was highly increased in the *NO66* transgenic mice when compared with nontransgenic controls (Figure 6). These results demonstrated an inverse correlation between BMSCs-derived adipogenic and osteogenic differentiation in transgenic mice.

## 4. Discussion

In this study, we generated an osteoblast-specific NO66 transgenic mouse model to investigate the role of NO66 in mammalian bone formation and homeostasis *in vivo*. Our data showed that the overexpression of NO66 from the 2.3 kB *Col1a1* promoter in cells of the osteoblast lineage has minimal impacts on the formation of both endochondral and intramembranous bones during the early developmental stages but affects bone formation and homeostasis in adult mice. This includes the decreased formation of cancellous bone tissues and fat accumulation in the bone marrow of long bones—an osteoporotic phenotype in adult mice.

The imbalance between adipogenesis and osteogenesis in the bone marrow is the predominant feature of osteoporosis [24, 25]. Numerous studies have demonstrated an inverse association between the accumulation of adipocytes and the loss of bone tissues in the marrow of aging patients with osteoporosis [4, 20–23, 26]. In our present study, the loss of cancellous bone tissues and the accumulation of BMF, which we observed in the long bones of adult *Col1a1-NO66* transgenic mice (Figures 4 and 5), resemble the features of age-related osteoporosis in humans. Therefore, our *Col1a1-NO66* transgenic mouse model constitutes a novel animal model for age-related osteoporosis.

The commitment and lineage differentiation of mesenchymal stem cells are controlled by specific transcription factors and signaling molecules [25, 27–29]. In our previous studies, we showed that deletion or overexpression of NO66 in the *Prx1*-expressing mesenchymal lineage affects differentiation of both osteogenic and chondrogenic progenitors, resulting in an abnormal skeleton and bone mass in adult animals [10, 11]. Here, we observed that transgenic mice with overexpression of NO66 in osteoblast lineage have minimal alteration in bone length (S Figure 3) or cartilage morphology (Figure 3(c)). This suggests that the osteoblast-specific overexpression of NO66 has little impact on chondrogenesis. The trabecular bone loss observed in the bone marrow of adult *Col1a1-NO66* transgenic mice is consistent with our previous *in vitro* study showing that NO66 is a negative regulator of osteoblast differentiation [9]. Given that the accumulation of BMF is often inversely correlated with bone loss in osteoporosis [4], it is likely that the increased fatty tissue observed in transgenic bone marrow is a secondary effect induced by an imbalance between osteogenesis and adipogenesis from BMSCs. However, we cannot rule out that certain signaling molecules, which are important for BMSC lineage commitment and differentiation, may also be affected by NO66 overexpression. The precise molecular mechanism underlying this process requires further investigation.

NO66 is a negative regulator of Osx activity [9–12]. NO66 knockdown in cultured preosteoblasts markedly increases osteoblast differentiation and expression of Osx target genes [9]. Similarly, genetic ablation of *NO66* in cells of the *Prx1*-expressing mesenchymal lineage accelerates osteoblast differentiation, expression of Osx-target genes *Bsp,* and *Oc* [10]. In contrast, mesenchymal overexpression of NO66 inhibits osteoblast differentiation, Osx-target gene expression, and bone formation [10, 11], highlighting the inverse relationship between the level of NO66 and the activity of Osx. However, a previous study showed that osteoblast-specific deletion of Osx driven by this 2.3 kB Col1a1 promoter does not cause clear bone defects in embryos and newborn pups but leads to osteopenia phenotypes in growing mice, including decreased BMD in the lumbar vertebra, thinner cortex of long bones, and immature trabecular bones [15]. Since NO66 acts as an inhibitor of Osx activity, in the transgenic mice overexpressing NO66 in the osteoblast lineage driven by the same 2.3 kB Col1a1 promoter, one would predict that the phenotypes of *Col1a1-NO66* mice should be similar to those of the osteoblast-specific Osx mutant mice. In agreement with this prediction, we did not observe overt defects in those *Col1a1-NO66* embryos and newborn mice when comparing with the nontransgenic controls, phenotypically and molecularly (Figure 3 and S Figure 2). Instead, we observed a significant decrease in bone volume fraction and BMD in the distal femoral bones of adult *Col1a1-NO66* mice (Figure 4). However, the cortical bones of our adult *Col1a1-NO66* mice did not appear thinner or more porous, unlike those observed in the osteoblast-specific Osx mutant mice [15], implicating a partial inhibition of Osx by NO66 overexpression. In addition, the predominant feature of our *Col1a1-NO66* mice is increased adipogenesis in the bone marrow of long bones, which was not shown in the osteoblast-specific Osx mutant mice [15]. This suggests that the phenotypic change observed in the long bones of the *Col1a1-NO66* mice may not simply be due to NO66-mediated attenuation of Osx activity and requires further examination.

## 5. Conclusions

Our studies using a *Col1a1-NO66* transgenic mouse model provide the first *in vivo* evidence that the chromatin modifier NO66 plays a crucial role in the regulation of aging bone formation and homeostasis. Our *Col1a1-NO66* transgenic mice constitute a new epigenetic animal model to study the chromatin alterations in the context of age-related osteoporosis, as well as a potential preclinical animal model for the evaluation of select novel drugs for the prevention and treatment of human osteoporosis.

## Data Availability

All data are fully available without restriction. All relevant data are within the manuscript and its supporting information files.

## Supplementary Materials

Supplementary Materials S Figure 1: immunofluorescence staining. S Figure 2: histological examination. S Figure 3: gross measurement of femur length. S Figure 4: histological examination. S Figure 5: histological examination.
