# Supplementary material for: Osteoblast-Specific Overexpression of Nucleolar Protein NO66/RIOX1 in Mouse Embryos Leads to Osteoporosis in Adult Mice
Source: J Osteoporos. 2023 Jan 10;2023:8998556. doi: 10.1155/2023/8998556 (PMC9845042; doi:10.1155/2023/8998556)
Supplement: Supplementary Materials — S Figure 1: immunofluorescence staining. S Figure 2: histological examination. S Figure 3: gross measurement of femur length. S Figure 4: histological examination. S Figure 5: histological examination. [file 8998556.f1.docx]

**Osteoblast-specific Overexpression of Nucleolar Protein NO66/RIOX1 In Mouse Embryos Leads to Osteoporosis in Adult Mice**

**Qin Chen,^1,2*^** **Krishna M. Sinha,^2,3^ Benoit de Crombrugghe^2^, Ralf Krahe^2*^**

^1^Department of Epigenetics and Molecular Carcinogenesis, ^2^Department of Genetics, ^3^Clinical Cancer Prevention, The University of Texas MD Anderson Cancer Center, Houston, TX 77030, USA

**Running title:** Overexpression of NO66/RIOX1 in osteoblasts leads to osteoporosis

* Corresponding authors

Email: [qchen1@mdanderson.org](mailto:qchen1@mdanderson.org) (QC); [rkrahe@mdanderson.org](mailto:rkrahe@mdanderson.org) (RK)

**Supplemental Figures**


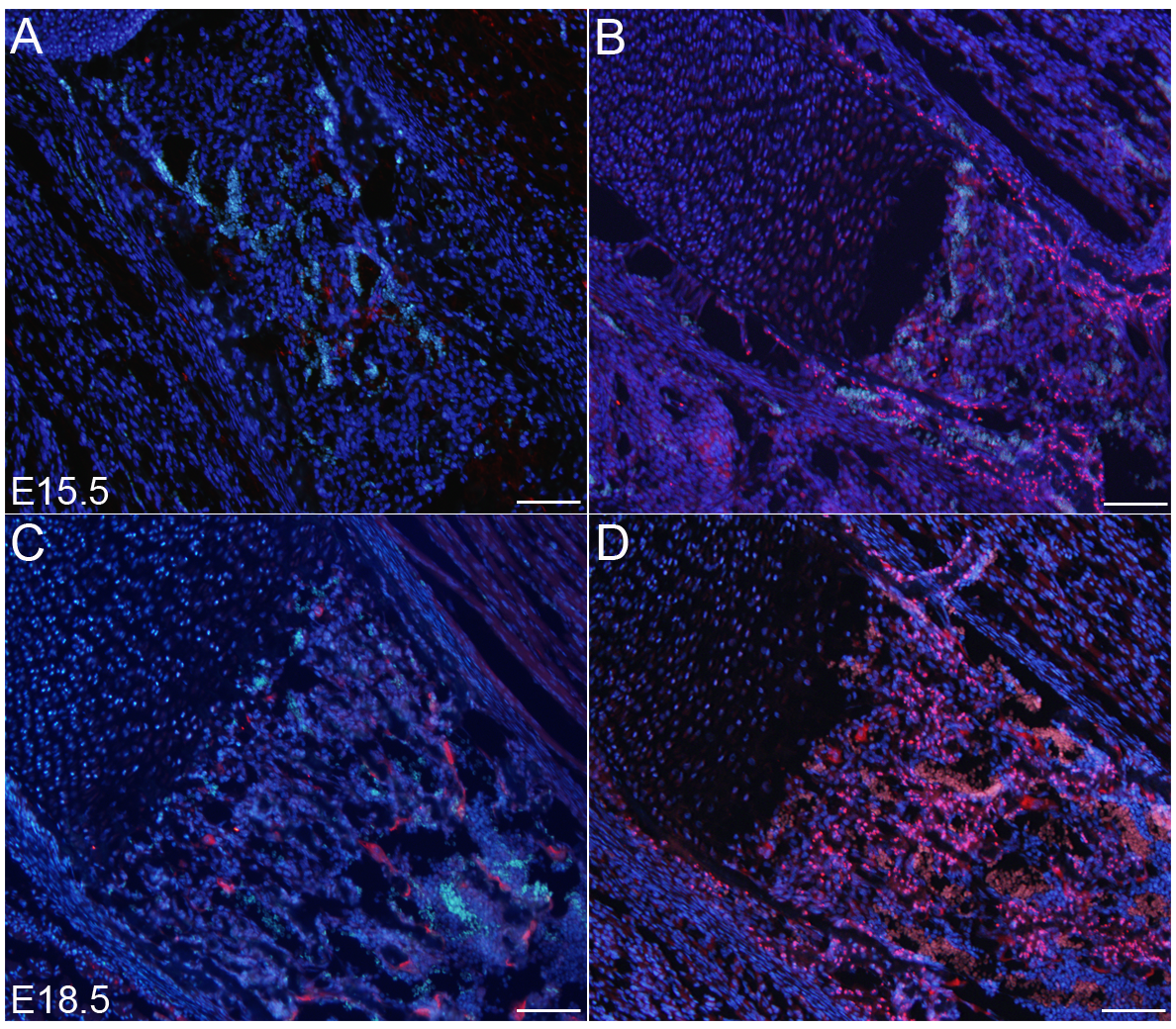


WT

Col1a1-NO66

**S Fig. 1.** Immunofluorescence staining. The femur sections of wild-type (WT) (**A**, **C**) and *Col1a1-NO66* transgenic (**B**, **D**) embryos at E15.5 and E18.5 were stained with anti-FLAG antibody (red). Cell nuclei were counterstained with DAPI (blue). The boxed areas indicate the location of the perichondrium/periosteum. White arrows in **B** and **D** point to cells expressing FLAG-tagged NO66 transgenic protein (pink nuclear staining).

**
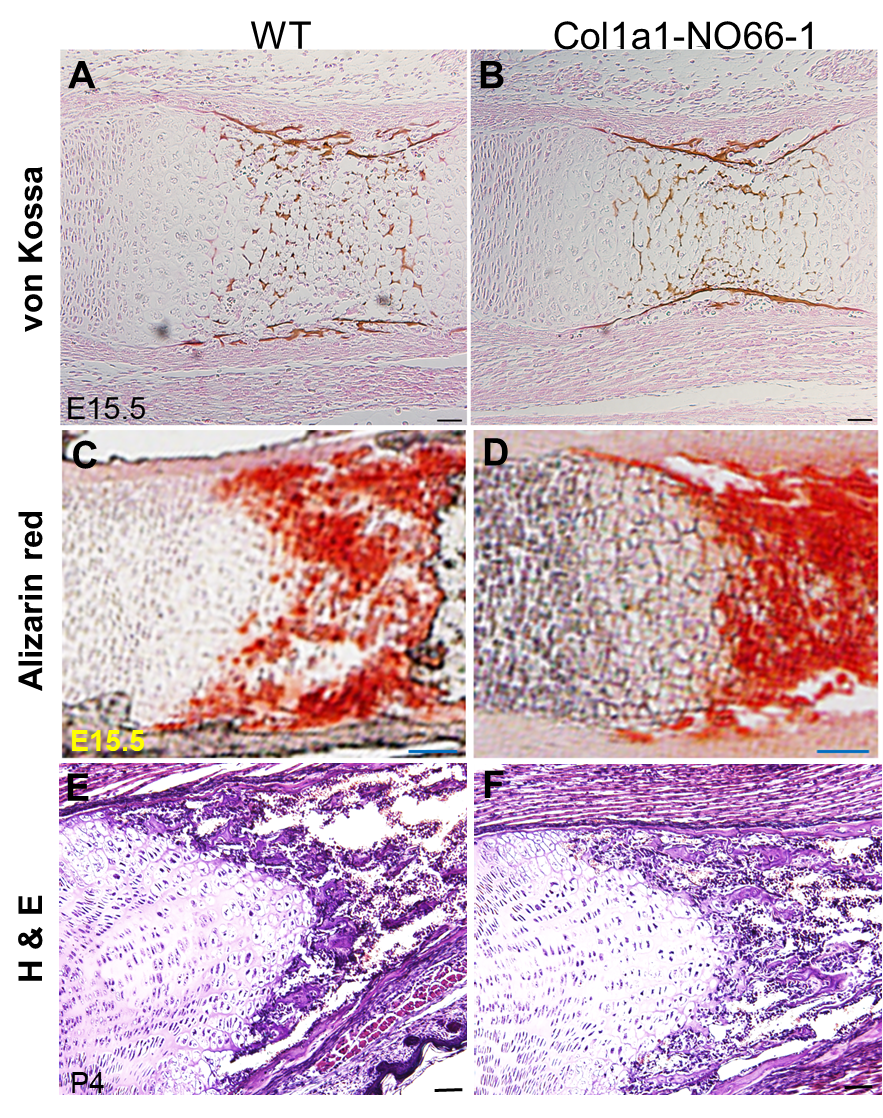
**

**S Fig. 2.** Histological examination. **A-D**, von Kossa (**A, B**) and Alizarin red (**C, D**) staining of the femur sections of wild type (WT) and NO66 transgenic (TG) embryos at E15.5. The brown (**A, B**) and red (**C, D**) staining signals indicate mineralized tissue. **E, F**, Hematoxylin & Eosin (H&E) staining of the femur sections of WT and TG mice at postnatal day 4 (P4).


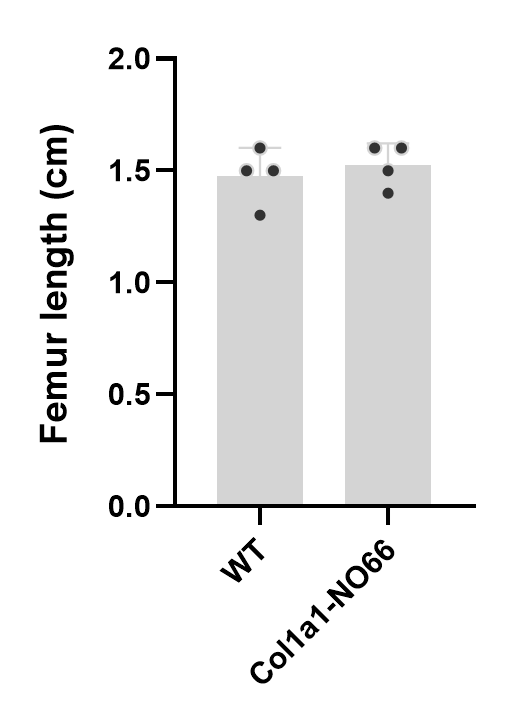


**S Fig. 3.** Gross measurement of femur length. The results of gross measurement in length of femur bones from three-month-old female wild type (WT) and NO66 transgenic mice (n=4) are shown.


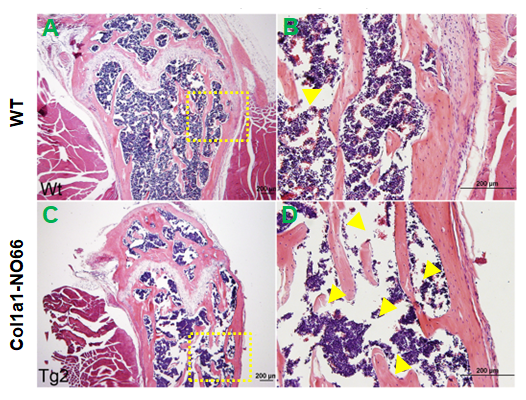


**S Fig. 4.** Histological examination. Hematoxylin & Eosin (H & E) staining of the distal femur sections of four-month-old male wild type (WT) (A, B) and transgenic mice (C, D). B, D are higher magnification of the boxed areas in A, C. Yellow arrowheads indicate fatty droplets within the bone marrow.


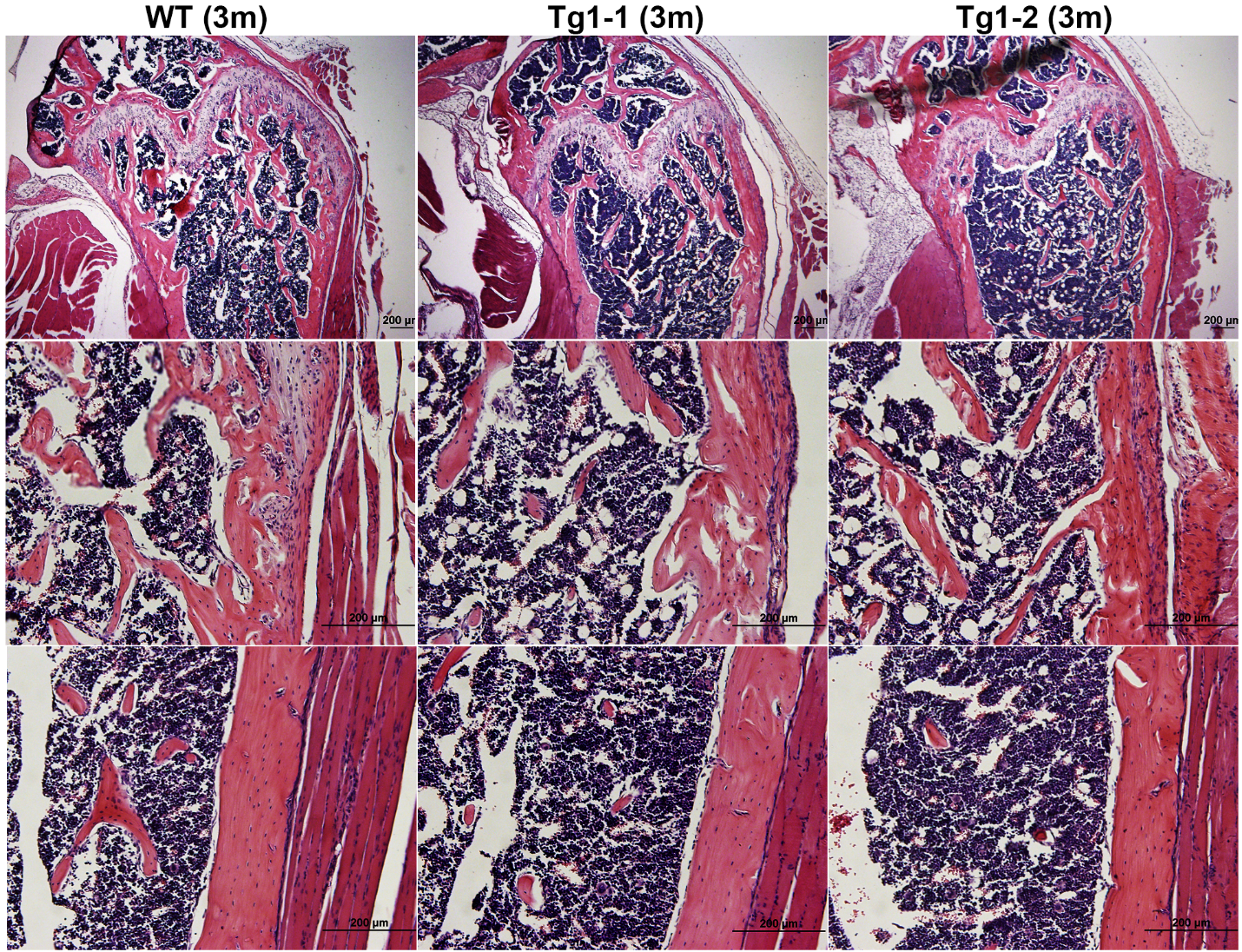


**WT**

**Col1a1-NO66-2**

**Col1a1-NO66-1**

**A**

**B**

**C**

**D**

**E**

**F**

**G**

**H**

**I**

**Tb**

**Gp**

**Cb**

**S Fig. 5**. Histological examination. H & E staining of the distal femur sections of three-month-old female wild type (WT) (**A, D, G**) and transgenic mice (from line 1) (**B, E, H**) and line 2 (**C, F, I**). **D-F** are higher magnification of the boxed areas in **A-C**; **G-I** are middle shaft images of femur sections shown in **A-C**; Yellow arrow heads indicate fatty droplets within the bone marrow. Gp, growth plate; Tb, trabeculae; Cb, cortical bone.
